# Fluorescent Probes for Biological Imaging

**DOI:** 10.1155/2016/3730486

**Published:** 2016-01-20

**Authors:** Xuanjun Zhang, Yupeng Tian, Jiangbo Yu, Zhen Yuan

**Affiliations:** ^1^Faculty of Health Sciences, University of Macau, Taipa, Macau; ^2^Department of Chemistry, Anhui University, Hefei 230601, China; ^3^Department of Chemistry, University of Washington, Seattle, WA 98195-1700, USA

Owing to the high sensitivity, high resolution, and the wealth of contrast mechanisms, fluorescence imaging is the most versatile and widely used visualization modality to study the structure and function of biological systems and the molecular process in living organisms without perturbing them.

Fluorescence molecular imaging is an evolving field of imaging sciences, which involves the development of microscopic techniques for live cell imaging at super resolution and macroscopic techniques to monitor molecular events in living organism. The breakthrough of super-resolution techniques allows researchers to obtain fluorescence images with a higher resolution than the diffraction limit. On the other hand, fluorescence imaging is also facing important challenges. Because the imaging requires exogenous probes to enhance imaging contrast or provide signal readout, the probe performance largely determines the detection limit and sensitivity. The intrinsic poor penetration of UV and visible light limits their broad applications in biology. Therefore, promising probes that exhibit high photostability, long fluorescence lifetime, strong absorption, and/or emission in the near-infrared (NIR) region are highly desirable.

Nanoparticle is a collection of atoms or molecules with much higher intensity of absorbance and emissions compared to small molecular probes, which can provide strong local contrast in biological imaging. Two-photon fluorescent probe simultaneously absorbs two infrared (IR) or NIR photons. Using IR or NIR light as excitation can minimize the light scattering and suppress the background signal, which allows imaging of living tissue up to about one millimetre in depth.

Applications of fluorescent probes for fluorescence molecular imaging are growing quickly for recording events from single live cells to whole animals with high sensitivity and accurate quantification. Such approaches have immense potential to track progression of metastasis, immune cell trafficking, stem cell therapy, transgenic animals, and even molecular interactions in living subjects, which represent the future and trends of optical molecular imaging technologies.

This special issue compiles several selected original and overview articles that range from design of contrast agents including fluorescent molecules and nanoparticles to biomedical molecular imaging and sensing with various applications. The editors believe that the selected work presented in this issue may provide useful information and promote further investigations on the development of novel fluorescent probes to be used for diagnosis and treatment of disorders and diseases as well as understanding the biological processes.

